# Upregulation of MMP-13 and TIMP-1 expression in response to mechanical strain in MC3T3-E1 osteoblastic cells

**DOI:** 10.1186/1756-0500-3-309

**Published:** 2010-11-17

**Authors:** Yongming Li, Lin Tang, Yinzhong Duan, Yin Ding

**Affiliations:** 1 Department of Orthodontics, College of Stomatology, The Fourth Military Medical University, Xi'an, Shaanxi Province 710032, PR China

## Abstract

**Background:**

Mechanical strain plays a significant role in the regulation of bone matrix turnover, which is mediated in part by matrix metalloproteinase (MMP)-13 and tissue inhibitors of matrix metalloproteinase (TIMP)-1. However, little is known about the correlation between mechanical strain and osteoblastic cell activities, including extracellular matrix (ECM) metabolism. Herein, we determined the effect of different magnitudes of cyclic tensile strain (0%, 6%, 12%, and 18%) on MMP-13 and TIMP-1 mRNA and protein expression in MC3T3-E1 osteoblasts. Furthermore, we employed specific inhibitors to examine the role of distinct signal transduction pathways known to mediate cellular responses to mechanical strain.

**Results:**

We identified a magnitude-dependent increase in MMP-13 and TIMP-1 mRNA and protein levels in response to mechanical strains corresponding to 6%, 12%, and 18% elongation. The strain-induced increases in MMP-13 and TIMP-1 mRNA expression were inhibited by PD098059 and cycloheximide, respectively.

**Conclusions:**

Our results suggest a mechanism for the regulation of bone matrix metabolism mediated by the differential expression of MMP-13 and TIMP-1 in response to increasing magnitudes of mechanical strain.

## Background

Bone is continuously remodeled throughout life in order to meet the functional demands of its physiological and mechanical environment [1-3]. Furthermore, active remodeling of alveolar bone must occur in order to cope with orthodontic force and mechanical loading generated during orthodontic tooth movement. This remodeling process requires a complex turnover of the bone extracellular matrix, which is mediated in part by matrix metalloproteinases (MMPs) and tissue inhibitors of matrix metalloproteinases (TIMPs)[4-7]. MMP-13, a member of the collagenase subgroup of MMP proteins, plays a key role in bone matrix degradation and is expressed highly in osteoblasts[3,8-12]. MMP-13 is likely to contribute to bone healing[13,14], bone development[15,16], and bone loss[17]. Previous studies also indicated that mRNA and protein levels of MMP-13 increase significantly following the application of orthodontic forces [18,19]. In contrast, TIMP-1 is an endogenous inhibitor of bone matrix degradation that binds tightly to active MMP-13, thereby downregulating MMP-13 activity[20-22].

The cellular response to mechanical strain is regulated by the type, frequency, magnitude, and duration of the mechanical strain imposed. In response to mechanical loading of bone tissue, osteoblasts exhibit changes in enzymatic activity and in protein production. The effect of mechanical force on the expression of MMPs has been demonstrated using various cell types [23-26]. However, few reports have examined the correlation between varying magnitudes of mechanical strain and osteoblastic cell activities, including extracellular matrix (ECM) metabolism. The relationship between mechanical strain and the expression of MMP-13 and TIMP-1 in osteoblasts is not known, particularly with respect to increasing magnitudes of mechanical strain.

In this study, we investigated the effect of different magnitudes of mechanical strain on MMP-13 and TIMP-1 expression in osteoblasts. MC3T3-E1 osteoblastic cells were subjected to 0%, 6%, 12%, or 18% elongation using the Flexercell Strain Unit, followed semi-quantitative reverse transcriptase-PCR (RT-PCR) and immunoblot analysis to determine MMP-13 and TIMP-1 mRNA and protein expression levels, respectively. Finally, we used specific inhibitors to determine the signal transduction pathways that regulate MMP-13 and TIPM-1 upregulation in response to mechanical strain.

## Methods

### MC3T3-E1 cell culture

Mouse osteoblastic MC3T3-E1 cells were obtained from the Center Laboratory for Tissue Engineering, College of Stomatology, Fourth Military Medical University, Xi'an, China. MC3T3-E1 cells were maintained at 37°C in a humidified atmosphere of 5% CO_2 _in α-modified Eagle's minimum essential medium (α-MEM: Sigma, St. Louis, MO, USA) containing 10% fetal bovine serum (FBS; JRH Biosciences, Lenexa, KS, USA), 32 U/ml penicillin G (Meiji Seika, Tokyo, Japan), 250 μg/ml amphotericin B (Nacalai Tesque, Kyoto, Japan), and 60 μg/ml kanamycin (Meiji Seika, Tokyo, Japan). After reaching 90% confluency, the cells were detached by treatment with 10% trypsin-EDTA (Sigma) and cultured for 24 h on six-well, flexible-bottomed plates (type I collagen-coated, Flex I; Flexcell International, McKeesport, PA, USA) at a density of 2 × 10^5 ^cells/well; the 10% FBS-containing medium was replaced with 1% FBS-containing medium prior to the application of mechanical strain.

### Application of strain force

Cells were plated onto six-well, flexible-bottomed plates at a density of 2 × 10^5 ^cells/well. After overnight incubation, the cells were nearly confluent and were subjected to mechanical strains of 6%, 12%, or 18% elongation at 6 cycles/min for 24 h using a Flexercell Strain Unit (FX 3000, Flexcell International), as described previously [27]. Control cells (0% elongation) were cultured on similar plates and were maintained in the same incubator without mechanical strain.

### RT-PCR

Semi-quantitative RT-PCR was used to determine the effect of mechanical strain on MMP-13 and TIMP-1 mRNA expression levels. Total RNA was isolated using an RNeasy mini kit (Qiagen, Chatsworth, CA, USA), followed by reverse transcription using random hexamers to generate cDNA. The cDNA was amplified using PCR primer pairs for MMP-13, TIMP-1, or the housekeeping gene glyceraldehyde-3-phosphate dehydrogenase (GAPDH) as a control. The following primer sets were used [3,4]: MMP-13 (445 bp RT-PCR product) sense 5'-GGTCCCAAACGAACTTAACTTACA-3', and MMP-13 antisense 5'-CCTTGAACGTCATCATCAGGAAGC- 3'; TIMP-1 (346 bp RT-PCR product) sense 5'-CCTTATACCAGCCGTTATAAGATCAAGAT-3', and TIMP-1 antisense 5'-GTCCACAAACAGTGAGTGTCACTC -3'; and GAPDH (983 bp RT-PCR product) sense 5'-GGTCGGTGTGAACGGATTTGG-3', and GAPDH antisense 5'-ATGTAGGCCATGAGGTCCACC-3'. PCR reactions for cDNA amplification included 2.5 mM dNTPs, 20 pmol/μl primers, and 5 U/μl Taq DNA polymerase. PCR amplification consisted of an initial denaturation step (94°C for 3 min), followed by thirty three-step cycles consisting of denaturation at 94°C for 30 s, annealing at a temperature optimized for each primer pair (MMP-13: 62°C, TIMP-1: 60°C, and GAPDH: 58°C) for 30 s, and extension at 72°C for 1 min; a final extension was carried out at 72°C for 10 min. The resulting PCR products were subjected to 2% agarose gel electrophoresis and were visualized by ethidium bromide staining. The relative intensities of the PCR products were measured using NIH Image software, and the results were normalized to the corresponding GAPDH mRNA levels.

### Immunoblot Analysis

Immunoblot analysis was performed using monoclonal antibodies against MMP-13 or TIMP-1 (Santa Cruz Biotechnology, Santa Cruz, CA, USA). Total protein lysates were collected from cells, and the protein concentration was determined using the Bradford assay (Bio-Rad); BSA was used to generate the standard curve. Approximately 50 μg of total protein was loaded in each well of a 10% SDS PAGE gel; GAPDH served as the control for protein loading. Proteins were transferred electrophoretically onto nitrocellulose membranes (Hybond C, Amersham), followed by incubation in PBS-T buffer [PBS (pH 7.5) buffer containing 5% (w/v) blocking reagent (Amersham) and 1.0% (w/v) Tween 20 (Sigma, St. Louis, MO, USA)]. Individual membranes were incubated overnight in PBS-T buffer containing antibodies against MMP-13 or TIMP-1, followed by a 1 h incubation with goat anti-rabbit IgG conjugated to horseradish peroxidase (ECL Western blotting analysis system, Amersham). Protein bands corresponding to MMP-13, TIMP-1, and GAPDH were visualized, and the density of the protein bands was analyzed.

### Enzyme-linked immunosorbent assay for the quantitative determination of active-MMP-13 and TIMP-1 concentrations

Conditioned media harvested from the cultured cells were used as samples. The concentrations of active form of MMP-13 and TIMP-1 in these supernatants were assayed using a comercially available enzyme-linked immunosorbent assay (ELISA) kits (R&D Systems, Minneapolis, MN). Each sample was assayed in triplicate. For active-MMP-13 quantification, the samples and standards were quantified fluorometrically with a fluorescence plate reader (MFX Microtiter1 Plate Fluorometer, Dynex technologies, Chantilly, VA, USA) with excitation wavelength set to 320 nm and emission wavelength set to 405 nm. For TIMP-1 quantification, the samples and standards were quantified with a microplate reader (MRX Microplate reader, Dynex Technologies, Chantilly, VA, USA) capable of measuring absorbance at 450 nm, with the correction wavelength set at 540 nm. The concentrations of active form of MMP-13 and TIMP-1 were determined using a standard curve and normalized to the total cell number in each sample.

### Inhibitors

We used specific inhibitors to identify signal transduction pathways that mediate the cellular response to tensile stress. We employed cycloheximide (10 μM) to inhibit *de novo *protein synthesis, indomethacin (10 μM) to inhibit cyclooxygenase (COX), genistein (20 μM) to inhibit tyrosine kinase activity, and PD098059 (10 μM) to specifically inhibit extracellular signal-related kinase (ERK). All inhibitors were purchased from Sigma (St. Louis, MO, USA), and the concentrations used correspond to effective doses reported previously [5,28]. MC3T3-E1 cells were pre-incubated in the presence of each inhibitor for 30 min, followed by the application of cyclic tensile strain at 18% elongation and 6 cycles/min in culture for 24 h. Total RNA was extracted from the cells, and MMP-13, TIMP-1, and GAPDH mRNA levels were determined by RT-PCR.

### Statistical analysis

We performed these experiments using samples from at least five different cell preparations, and reproducibility was confirmed by using the same cell sample in triplicate. Values were calculated as means ± standard deviation (SD). Some data were subjected to multiple measurement analyses of variance (ANOVA), and a Student's t test was used to determine differences between the groups tested. *P *values of less than 0.05 were considered significant.

## Results

**Figure 1:**
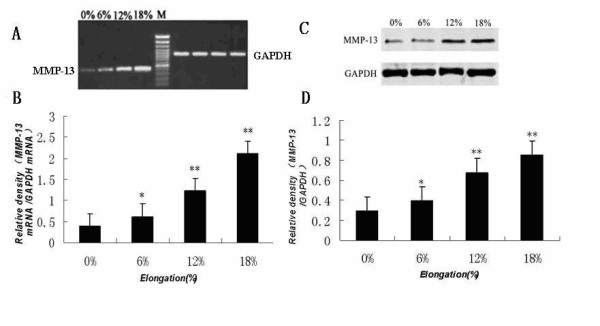
**Effect of increasing tensile strain on MMP-13 mRNA and protein levels in mouse osteoblastic MC3T3-E1 cells**. Cells were seeded at a density of 2 × 10^5 ^cells/well on Flex I culture plates and cultured in α-MEM medium supplemented with 10% FBS for 24 h. The cells were then loaded with (6%, 12%, 18%) or without (0%) cyclic tensile strain at the indicated percent elongation at 6 cycles/min using a Flexercell strain unit for 24 h. The levels of MMP-13 mRNA and protein were determined by RT-PCR and immunoblot analysis, respectively, as described in the Materials and methods. (A) Agarose gel electrophoresis of the RT-PCR products using specific primers for MMP-13 or GAPDH. (B) MMP-13 mRNA expression levels normalized to GAPDH. (C) Immunoblot analysis of MMP-13 and GAPDH. (D) MMP-13 protein expression levels normalized to GAPDH. The results shown are means ± SD of five independent experiments. Significant differences from the 0% culture are shown by ANOVA and Student's t test (*P < 0.05, ** P < 0.01).

Figure 1 shows how different magnitudes of strain affect MMP-13 mRNA and protein expression levels in the MC3T3-E1 cells. The expression of MMP-13 mRNA and protein were increased significantly and in a magnitude-dependent manner by mechanical strain with 6%, 12% or 18% elongation in comparison with the control (0% elongation) cultures.

**Figure 2:**
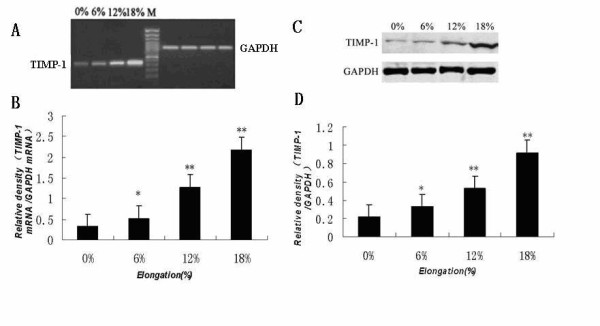
**Effect of increasing tensile strain on TIMP-1 mRNA and protein levels in mouse osteoblastic MC3T3-E1 cells**. Cells were seeded at a density of 2 × 10^5 ^cells/well, cultured for 24 h, and were then loaded with (6%, 12%, 18%) or without (0%) tensile strain at the indicated percent elongation at 6 cycles/min using a Flexercell strain unit for 24 h. TIMP-1 mRNA and protein levels were determined by RT-PCR and Western blot analysis, respectively, as described in the Materials and methods. (A) Agarose gel electrophoresis of the RT-PCR products using primers specific for TIMP-1 or GAPDH. (B) TIMP-1 mRNA expression levels normalized to GAPDH mRNA levels. (C) Immunoblot analysis of TIMP-1 and GAPDH. (D) TIMP-1 protein expression levels normalized to GAPDH. The results shown are means ± SD of five independent experiments. Significant differences from the 0% culture are shown by ANOVA and Student's t test (*P < 0.05, ** P < 0.01).

Figure 2 shows how different magnitudes of strain affect the TIMP-1 mRNA and protein expression levels in the MC3T3-E1 cells. Mechanical strain at 6%, 12% or 18% elongation caused a significant magnitude-dependent increase in TIMP-1 mRNA and protein expression compared to that in control (0% elongation) cultures.

**Figure 3:**
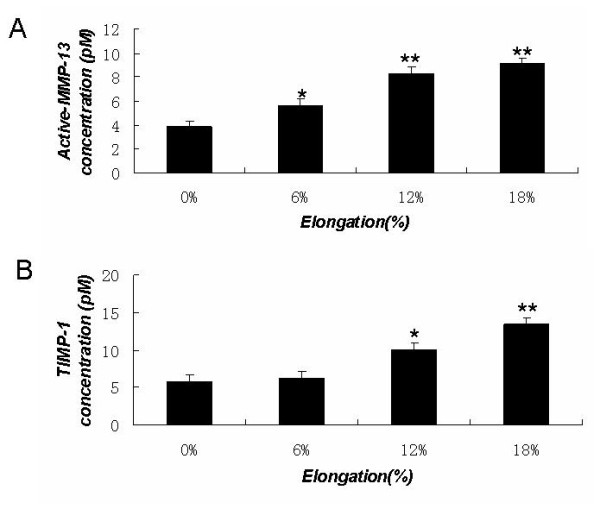
**Active-MMP-13 and TIMP-1 concentrations in conditioned medium induced by mechanical strain in MC3T3-E1 cells**. Conditioned media harvested from the cultured cells were used as samples. The concentrations of active form of MMP-13 and TIMP-1 in these supernatants were assayed using a comercially available enzyme-linked immunosorbent assay (ELISA) kits, as described in the Materials and methods. (A) The concentrations of active form of MMP-13. (B) The concentrations of TIMP-1. The results shown are means ± SD of five independent experiments. Significant differences from the 0% culture are shown by ANOVA and Student's t test (*P < 0.05, ** P < 0.01).

The elevated expressions of MMP-13 and TIMP-1 in the stretched cells were further confirmed by ELISA for quantifying active-MMP-13 and TIMP-1 concentrations in conditioned medium from these cells. Active-MMP-13 and TIMP-1 concentrations in conditioned medium induced by mechanical strain in MC3T3-E1 cells also increased significantly in a magnitude-dependent manner compared to that in the control (See figure 3).

**Figure 4:**
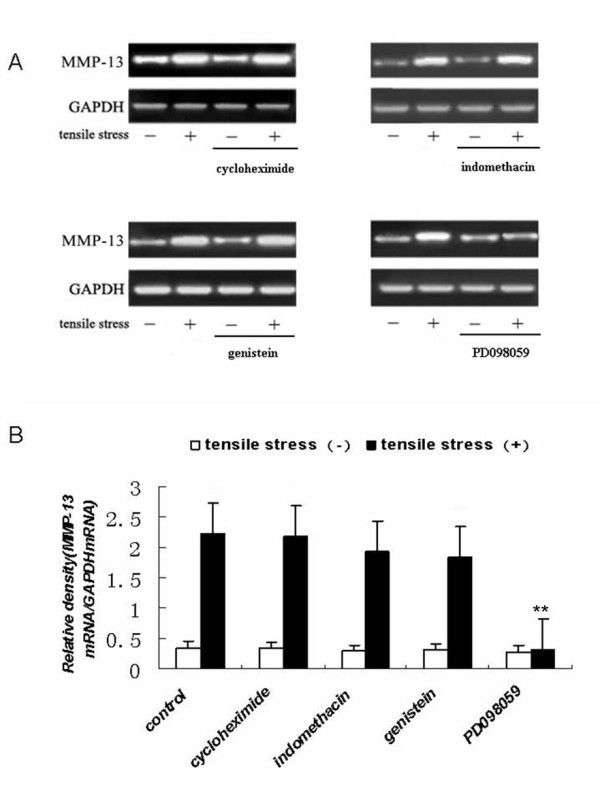
**Effects of inhibitors on tensile strain-induced expression of MMP-13 mRNA in MC3T3-E1 cells**. Cells were seeded at a density of 2 × 10^5 ^cells/well on Flex I culture plates and grown in α-MEM medium supplemented with 10% FBS for 24 h. Cells were then cultured in medium containing cycloheximide (10 μM), indomethacin (10 μM), genistein (20 μM), PD098059 (10 μM), or vehicle (control) for 30 min. The cells were cultured with (+) or without (-) loading with tensile strain at 18% elongation at 6 cycles/min for 24 h. Total RNA was extracted from the cells, and expression levels of MMP-13 mRNA were determined by RT-PCR as described in the Materials and methods. (A) Agarose gel electrophoresis of MMP-13 and GAPDH RT-PCR products. (B) MMP-13 mRNA expression levels normalized to GAPDH. The results shown are means ± SD of five independent experiments. **Indicates a significant difference from the strain (+) culture (P < 0.01)as determined by Student's t test.

**Figure 5:**
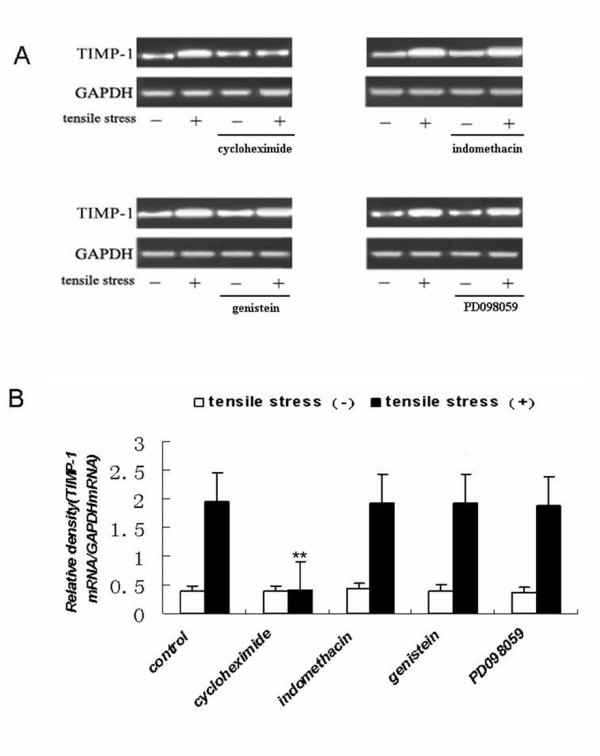
**The effects of inhibitors on tensile strain-induced expression of TIMP-1 mRNA in MC3T3-E1 cells**. Cells were seeded at a density of 2 × 10^5 ^cells/well on Flex I culture plates and were cultured in α-MEM medium supplemented with 10% FBS for 24 h. They were then cultured in medium containing cycloheximide (10 μM), indomethacin (10 μM), genistein (20 μM), PD098059 (10 μM), or vehicle (control) for 30 min. The cells were cultured with (+) or without (-) loading with tensile strain at 18% elongation at 6 cycles/min for 24 h. Total RNA was isolated, and TIMP-1 and GAPDH mRNA levels were determined by RT-PCR. (A) Agarose gel electrophoresis of the TIMP-1 and GAPDH RT-PCR products. (B) TIMP-1 mRNA levels normalized to GAPDH. The results shown are means ± SD of five independent experiments. **Indicates a significant difference from stress (+) culture as determined by Student's t test (P < 0.01).

To determine whether the stretch-induced increases in MMP-13 and TIMP-1 mRNA expression were dependent on de novo protein synthesis, cyclooxygenase activity, tyrosine kinase activity, or extracellular signal-related kinase activity, MC3T3-E1 cells were treated with specific inhibitors, including cycloheximide, indomethacin, genistein, or PD098059, respectively. We found that only the MEK inhibitor PD098059 was capable of blocking the strain-induced upregulation of MMP-13 mRNA (See figure 4). In contrast, the signaling pathway linked to the induction of TIMP-1 mRNA expression was blocked only by cycloheximide (See figure 5).

## Discussion

It has been demonstrated that matrix metalloproteinases (MMPs) and tissue inhibitors of metalloproteinases (TIMPs) regulate matrix degradation. The balance between the activities of MMPs and TIMPs is believed to determine the rate of this process. Previous studies indicate that MMP-13 plays a key role in bone remodeling and mechanical strain can affect the expression of MMP-13 mRNA in osteoblasts[3]. As we know the effects of mechanical strain on cells are dependent on the magnitude, duration, and frequency of mechanical strain, however, to date, very few reports have shown the effects of different magnitudes of mechanical strain on the mRNA and protein expression levels of MMP-13 and TIMP-1 in osteoblasts. The molecular biology mechanism of the regulation of extracellular matrix metabolism of osteoblasts under mechanical strain is not clear that progressive study should be carried out to clarify how different magnitudes of mechanical strain affect the expression of MMP-13 and TIMP-1 in osteoblasts. Yang et al. [3] reported previously that MMP-13 mRNA levels exhibited a time-dependent increase in MC3T3-E1 cells following the application of mechanical strain corresponding to 8% elongation. But, the correlation between varying magnitudes of mechanical strain and the expression of MMP-13 in osteoblasts is not known. The present study demonstrates that MC3T3-E1 cells subjected to 6%, 12%, or 18% elongation exhibit a magnitude-dependent increase in MMP-13 mRNA and protein levels (See figure 1). Active-MMP-13 concentration in conditioned medium induced by mechanical strain in MC3T3-E1 cells also increased significantly in a magnitude-dependent manner compared to that in the control (See figure 3). Several lines of evidence suggest that low levels of mechanical strain (1.8% - 6% elongation) generate potent anti-inflammatory signals, whereas high levels of mechanical strain (12% - 18% elongation) generate an inflammatory signal and induce PGE_2 _and IL-1β, both implicated in matrix degradation[29-32]. In the present study, we found that the increase in MMP-13 expression was magnitude-dependent. Furthermore, mechanical strain corresponding to 6%, 12%, or 18% elongation led to a magnitude-dependent increase in TIMP-1 mRNA and protein expression (See figure 2). TIMP-1 concentration in conditioned medium induced by 12%, or 18% elongation mechanical strain increased significantly compared to that in the control (See figure 3). Collectively, these results suggest that the increase in MMP-13 levels observed in osteoblastic cells under mechanical strain levels higher than those found under normal physiological conditions is accompanied by a corresponding increase in TIMP-1. TIMP-1 may in turn bind to activated MMP-13, thereby inhibiting MMP-13 activity and suppressing the degradation of bone matrix as part of the body's defense system. Our present findings may help to explain the lack of pathological inflammatory changes during orthodontic tooth movement when mechanical strain loads the alveolar bone. It appears that the essential functions of osteoblasts in bone remodeling are affected by the magnitude of mechanical strain and that cellular responses to mechanical strain are crucial to maintaining homeostasis and adapting to the bone environment.

The molecular mechanisms translating mechanical strain into a signal that activates increased gene expression are complicated. Mechanotransduction pathways may initiate signal transduction through many possible mechanisms including integrins, receptor tyrosine kinases, ion channel, gap junction, membrane fluidity, etc. To determine whether the stretch-induced increases in MMP-13 and TIMP-1 mRNA expression were dependent on *de novo *protein synthesis, cyclooxygenase activity, tyrosine kinase activity, or extracellular signal-related kinase activity, MC3T3-E1 cells were treated with specific inhibitors, including cycloheximide, indomethacin, genistein, or PD098059, respectively. We found that only the MEK inhibitor PD098059 was capable of blocking the strain-induced upregulation of MMP-13 mRNA (See figure 4). This result indicates that MMP-13 induction is mediated by signaling molecules related to extracellular signal-regulated kinase (ERK), which is supported by a previous study[3]. Therefore, the ERK-MAPK pathway is likely to contribute to the strain-induced increase in MMP-13 mRNA expression. In contrast, the signaling pathway linked to the induction of TIMP-1 mRNA expression was blocked by cycloheximide (See figure 5), indicating that strain-induced up-regulation of TIMP-1 mRNA required *de novo *protein synthesis, and suggesting that the strain-induced upregulation of TIMP-1 mRNA is not the primary response; rather, TIMP-1 upregulation required the synthesis of an as yet unidentified protein(s).

## Conclusions

In conclusion, the application of different magnitudes of cyclic tensile strain (0%, 6%, 12%, or 18%) induced a magnitude-dependent increase in MMP-13 and TIMP-1 expression in cultured osteoblasts. Furthermore, we provide evidence that PD098059 and cyclohexamide treatment suppressed the strain-induced upregulation of MMP-13 and TIMP-1 expression, respectively. These results indicate that MMP-13 and TIMP-1 expression are differentially regulated in response to increasing magnitudes of mechanical strain in osteoblasts. This effect may regulate bone matrix metabolism, and suggests novel mechanisms through which osteoblasts may respond and adapt to mechanical strain, including occlusal and orthodontic forces, in order to maintain homeostasis and adapt to the bone environment.

## Competing interests

The authors declare that they have no competing interests.

## Authors' contributions

YL, LT and YD designed the study. YL and LT performed the laboratory work. YL and LT performed data analysis. Interpretation of data and writing of the manuscript were done by YL, LT and YD. All authors read and approved the manuscript.
